# Family history of substance use disorder and parental impulsivity are differentially associated with neural responses during risky decision-making

**DOI:** 10.3389/fnimg.2023.1110494

**Published:** 2023-06-21

**Authors:** Joseph Aloi, Elizabeth Kwon, Tom A. Hummer, Kathleen I. Crum, Nikhil Shah, Lauren Pratt, Matthew C. Aalsma, Peter Finn, John Nurnberger, Leslie A. Hulvershorn

**Affiliations:** ^1^Department of Psychiatry, Indiana University School of Medicine, Indianapolis, IN, United States; ^2^Department of Public Health, Baylor University, Waco, TX, United States; ^3^Stark Neurosciences Research Institute, Indiana University School of Medicine, Indianapolis, IN, United States; ^4^Department of Pediatrics, Indiana University School of Medicine, Indianapolis, IN, United States; ^5^Department of Psychology, Indiana University-Bloomington, Bloomington, IN, United States

**Keywords:** impulsivity, risky decision-making, fMRI, adolescent, substance use disorder

## Abstract

**Background:**

Risky decision-making is associated with the development of substance use behaviors during adolescence. Although prior work has investigated risky decision-making in adolescents at familial high risk for developing substance use disorders (SUDs), little research has controlled for the presence of co-morbid externalizing disorders (EDs). Additionally, few studies have investigated the role of parental impulsivity in offspring neurobiology associated with risky decision-making.

**Methods:**

One-hundred twenty-five children (28 healthy controls, 47 psychiatric controls with EDs *without* a familial history of SUD, and 50 high-risk children *with* co-morbid EDs with a familial history of SUD) participated in the Balloon Analog Risk Task while undergoing functional magnetic resonance imaging. Impulsivity for parents and children was measured using the UPPS-P Impulsive Behavior Scale.

**Results:**

We found that individuals in the psychiatric control group showed greater activation, as chances of balloon explosion increased, while making choices, relative to the healthy control and high-risk groups in the rostral anterior cingulate cortex (rACC) and lateral orbitofrontal cortex (lOFC). We also found a positive association between greater activation and parental impulsivity in these regions. However, within rACC, this relationship was moderated by group, such that there was a positive relationship between activation and parental impulsivity in the HC group, but an inverse relationship in the HR group.

**Conclusions:**

These findings suggest that there are key differences in the neurobiology underlying risky decision-making in individuals with EDs with and without a familial history of SUD. The current findings build on existing models of neurobiological factors influencing addiction risk by integrating parental factors. This work paves the way for more precise risk models in which to test preventive interventions.

## 1. Introduction

Adolescents and adults with substance use disorders show altered decision-making in several domains, such as preferences for short-term gains (Bechara et al., 2002), selecting riskier options (Lane and Cherek, 2000), difficulty valuing potential outcomes (Aloi et al., 2021a), as well as decreased activation to rewarding outcomes (Aloi et al., 2020, 2021b). It has been suggested that impulsivity during decision making partly mediates the risk for developing substance use behaviors in adolescence (Schneider et al., 2012). Thus, impairments in both impulse control and decision making are crucial predictors of substance use disorder (SUD) risk (Crews and Boettiger, 2009). Although prevention programs have been developed for children at risk of SUDs, effect sizes are small and programs do not directly address decision-making (Sussman et al., 2004). It is therefore important to inform interventions aimed at SUD prevention through a greater understanding of the neurobiology of risk-relevant processes.

Evidence from twin studies indicates that impulsive behaviors are heritable (Tiego et al., 2020), and that impulsivity may play a role in the association between family history of SUD and offspring SUD development (Mitchell and Potenza, 2014). Impulsivity has been suggested as an intergenerationally transmitted intermediate phenotype linking dopamine receptor polymorphisms and substance use behaviors (Gorwood et al., 2012). Variation in risky decision making has also been associated with genetic factors (Tuvblad et al., 2013) (35–46% of variance explained), suggesting that the well-established heritability of SUDs is likely multifactorial, similar to other psychiatric conditions (Hicks et al., 2004). Despite this genetic literature, prior studies that have examined reward- and decision-processing neural circuits in samples of children at elevated risk for SUDs have not accounted for parental impulsivity (and have often ignored the effects of childhood impulsivity). Therefore, the role of intergenerational transmission of SUD risk factors, particularly as reflected in the developing brain, is poorly understood.

Impulsivity and decision-making difficulties have been clinically operationalized in childhood as externalizing disorders, such as attention-deficit/hyperactivity disorder (ADHD), conduct disorder (CD), or oppositional defiant disorder (Moss and Lynch, 2001; ODD). The phenomenology of externalizing disorders has some overlap with SUDs, hypothetically via greater impulsivity and impairments in decision-making (Martel et al., 2017). Consistent with this phenomenological overlap, neuroimaging work has shown that individuals with externalizing disorders show neural dysfunction similar to adolescents with SUDs during decision-making tasks, specifically in the rostral anterior cingulate cortex (rACC), lateral orbitofrontal cortex (lOFC), and anterior insular cortex (White et al., 2013, 2016a,b) (aIC). Moreover, externalizing disorders show familial transmission via the heritability of impulsivity (Hicks et al., 2004), similar to substance use behaviors (Arcos-Burgos et al., 2012). However, little prior work investigating the neurobiology of familial risk factors of SUD has controlled for other risk factors, such as the presence of co-morbid externalizing disorders.

Family history of SUD confers risk via abnormalities in reward processing and decision-making neural circuits (Ivanov et al., 2012). During a Balloon Analog Risk Task (BART), children with co-morbid externalizing disorders and paternal SUD history showed greater activation to negative outcomes within ventromedial prefrontal cortex (vmPFC), ACC, right inferior frontal gyrus (iFG)/anterior insular cortex (aIC), and ventral striatum compared to healthy controls (Hulvershorn et al., 2015). Yet, it should be noted that adolescents who have already transitioned to substance use behaviors show greater activation to risky decisions within parietal cortices, striatum, and dorsal ACC (Claus et al., 2017). Prior work in twin data has also demonstrated that activity within these brain regions are both heritable and related to impulsivity (Rao et al., 2018). Moreover, children with co-morbid ADHD and familial history (FH) of SUDs showed reduced frontal activation and increased striatal activation to anticipation of reward during an anticipation, conflict, reward (ACR) task compared to ADHD-only and typically developing (TD) groups (Ivanov et al., 2019). Children with co-morbid ADHD and FH of SUDs showed greater learning rates (i.e., they give more weight to the most recent trials when updating expected values) and reduced reaction times during an anticipation, conflict, reward (ACR) task compared to ADHD-only and TD control groups (Parvaz et al., 2018). However, it would be expected that children with FH of SUDs would have one or more parent(s) with greater impulsivity (Jentsch et al., 2014). Therefore, it is possible that some of these findings in children with co-morbid ADHD and FH of SUDs (Parvaz et al., 2018; Ivanov et al., 2019) are driven by intergenerational transmission of impulsivity from parents to children (whether by genetic or environmental mechanisms). No studies to date have investigated the association between parental impulsivity and neural alterations in decision-making in children.

The purpose of this study was to address these gaps in the literature by (i) investigating differences in neural functioning during risky decision-making in children with externalizing disorders with and without family histories of SUD; and (ii) investigate the relationship between parental impulsivity and child neural functioning during risky decision-making. We used the BART (Hulvershorn et al., 2015) to index risky decision-making, and measured neural responses during the choice and outcome phases of this task in three groups: (i) Healthy Controls (“HC”), (ii) Children with externalizing disorders, but no FH of SUDs (Psychiatric Controls, “PC”), and (iii) Children with comorbid externalizing disorders and FH of SUDs (High Risk, “HR”). Specifically, prior work has shown that high-risk children had greater activation to negative outcomes during the BART task within inferior frontal gyrus (iFG), aIC, ACC, and ventral striatum (Hulvershorn et al., 2015), compared to healthy control children. We therefore hypothesized that in this current study, HR children would show greater activation to negative outcomes of risky decisions within these same brain regions during outcome phase of the BART compared to a novel group of PC children. Since these brain regions were identified using the BART task in prior work (Hulvershorn et al., 2015), we used the BART to index risky decision-making for the current study. Moreover, given the heritability of brain activity during the BART (Rao et al., 2018), we hypothesized that parental impulsivity would be associated with greater activation to negative outcomes of risky decisions. However, it is worth noting that others have found hypo-responsivity in brain regions associated with valuation of stimuli and orchestrating the attentional response to these stimuli—such as aIC, lOFC, and rACC—during the choice phase of decision-making tasks (Crowley et al., 2010). Based on those findings, we hypothesized that HR children would also show reduced activation to risky choices during the choice phase of the BART, relative to other groups. Likewise, we also hypothesized that parental impulsivity would be associated with reduced activation during the choice phase of the BART.

## 2. Methods

### 2.1. Sample

One hundred and ninety-two English-speaking, right-handed, 11–12 year-old participants were recruited in a US community-based sample as part of an ongoing longitudinal study (Dir et al., 2019, 2020; Kwon et al., 2021). After consent and assent, to determine eligibility, onsite interviews were conducted in-person at study facilities by child mental health clinicians using a DSM-5 (American Psychiatric Association, 2013) modified version of the K-SADS-PL (Kaufman et al., 1997). Depending on the diagnoses and family history of SUDs, participants were divided into three groups: the healthy control (HC) group included adolescents with no externalizing psychopathologies and no history of SUDs in the biological parents or other first-degree relatives. The psychiatric control (PC) group included those who met current DSM-5 (Diagnostic and Statistical Manual of Mental Disorders) criteria for any sub-type of ADHD and a disruptive behavior disorder (DBD; either ODD, CD, or DBD-unspecified) and no history of SUDs in the biological parents or other first-degree relatives. The high risk (HR) for the development of SUDs group had identical externalizing psychopathologies as the PC group but also had a biological father with a current or past DSM-5 SUD (excluding isolated tobacco or alcohol use disorders in the absence of drug use disorders) and another 1st or 2nd degree family member with a SUD history to capture the full spectrum of SUDs in these parents.

Of the 192 initial participants, 39 were in the HC group, 70 were in the PC group, and 83 were in the HR group. Of the 192 participants, 11 withdrew prior to scanning, and then 55 participants were excluded for scan quality (e.g., excessive movement, behavioral artifacts, incomplete scanning data), resulting in a final sample of 125 participants (28 HC, 47 PC, and 50 HR).

Of the 125 participants included in the study, 53 participants lived with both biological parents, 63 lived with their biological mother but not their biological father, 3 lived with their biological father but not their biological mother, and 4 lived with neither biological parent. Parental SUDs were evaluated using the Semi Structured Assessment for the Genetics of Alcoholism (SSAGA) (Hesselbrock et al., 1999) conducted with both biological parents wherever possible. In all cases, SSAGA interviews were attempted with both biological parents; in most cases, however, one parent was unavailable so an informant SSAGA interview was obtained with the available parent or guardian. Parents, however, were not present during the interviews with children. Exclusion criteria for all groups included past or present psychotic symptoms; autism spectrum disorder; current depression or mania; substance use; neurological problems; debilitating medical conditions; estimated Full-Scale IQ < 80 on the Wechsler Abbreviated Scale of Intelligence (Wechsler, 2011); routine MRI contraindications; and use of any psychopharmacologic medications within the last 2 weeks, apart from psychostimulants, which were held on study days. Exclusion criteria were verified by parent report. Also, as *in utero* exposure to substances could directly influence brain development, adolescents with parent-reported prenatal drug exposure were excluded from the sample.

### 2.2. Measures

#### 2.2.1. BART task

**Figure 1:**
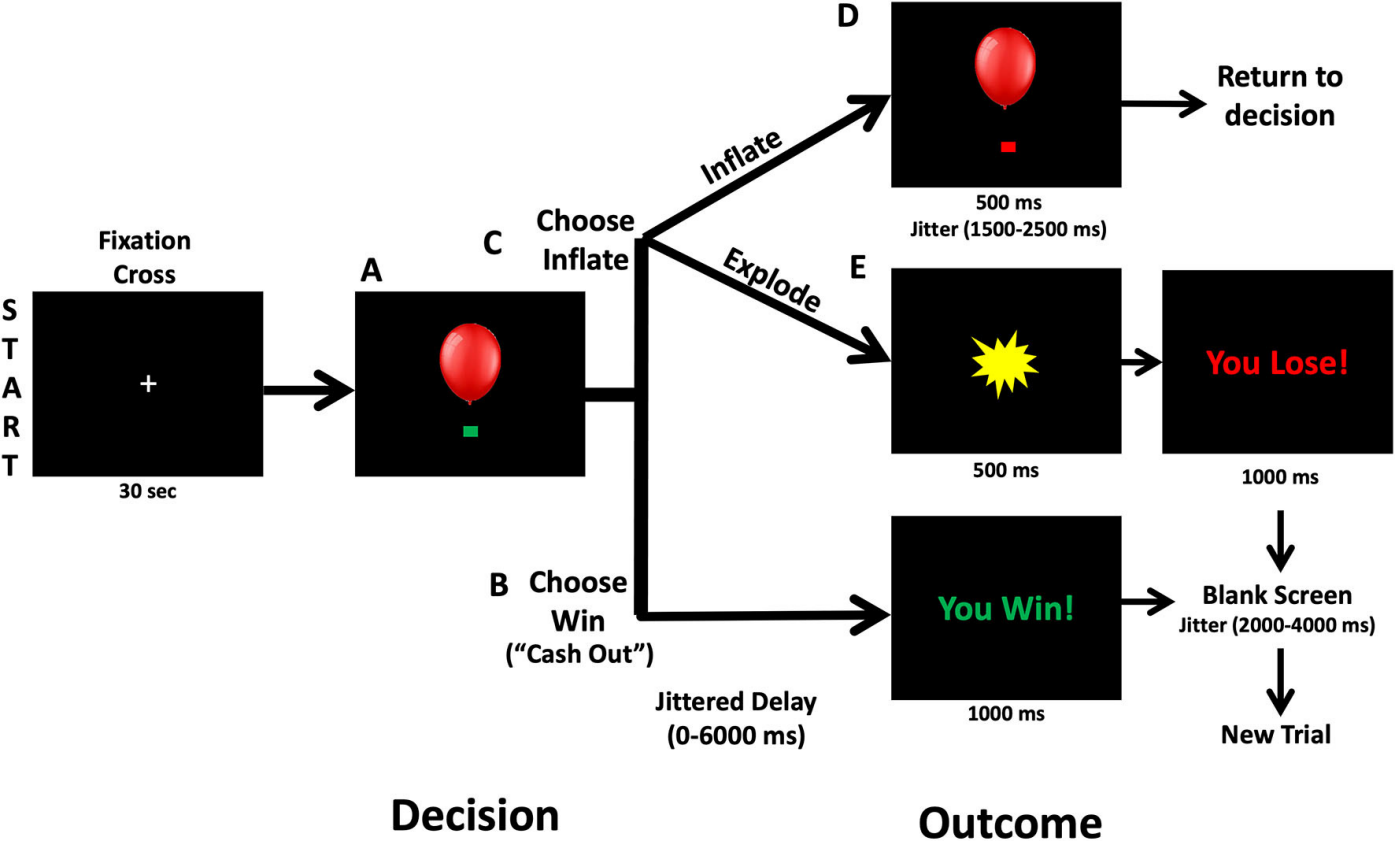
Balloon analog risk task. At the beginning of each trial, a balloon and green decision cue are shown on the screen. **(A)** Participants can decide to inflate the balloon or cash out and take the accumulated wager via a button press. **(B)** Following Choose Win decisions, there is a 0–6,000 ms jitter followed by a screen that says “You Win!” for 1,000 ms. Then a 2,000–4,000 ms fixation is shown prior to starting a new balloon trial. **(C)** Following a decision to inflate the balloon (Choose Inflate), the balloon can either explode or inflate. **(D)** If the balloon inflates, an inflated balloon is shown for 1,500–2,500 ms before returning to the decision period and allowing another choice. **(E)** If the balloon explodes, participants are shown an explosion for 1,500 ms and then a fixation cross for 2,000–4,000 ms.

A version of the BART modified for use in MRI scanning (Hulvershorn et al., 2015) was administered during MRI scanning to measure brain activation during risky decision-making (Figure 1). In the BART task, participants decide whether to virtually inflate a balloon to risk monetary reward that increase with each successive balloon inflation or to bank the amount of money they have already made and start a new balloon. Participants were shown a simulated balloon and pressed one of two buttons to either inflate the balloon (Choose Inflate), risking cash rewards that increase with the size of the balloon, or stop inflating and bank the accumulated money (Choose Win) and start a new balloon. If participants choose to inflate, the balloon image either inflates and increases the accumulated rewards (Outcome Inflate) or explodes and lose accumulated money (Outcome Explode). Except the first inflation, explosions could occur at any balloon size, but the risk of explosion increases as the balloon size increases. Following the explosion, a new balloon is started. When participants choose to win the money, accumulated cash rewards are banked, and a new balloon is presented. Over 38 min runs, participants could complete as many balloons as possible. A maximum of 12 inflations were possible per balloon. Before the administration of the BART, participants practiced and understood that they would be paid with cash after the scan based on their performance. Measures of risk taking included the following: the mean number of inflations on unexploded balloons (average adjusted pump), total number of balloon explosions (Outcome Explode), number of balloon inflations (Outcome Inflate), mean Choose Win wager, the number of Choose Win decisions, the number of pump (Choose Inflate). Lastly, we measured reaction time, which is the time duration that participants took to make a choice and push the button for Choose Inflate and Choose Win separately.

#### 2.2.2. Parent and child impulsivity

Parent and child self-reported impulsivity was measured using Urgency, Premeditation, Perseverance, Sensation Seeking, and Positive Urgency (UPPS-P) Impulsive Behavior Scale (Whiteside and Lynam, 2001) and the UPPS-P-C (Zapolski et al., 2010; validated for children aged 7–13), respectively. Parental impulsivity was collected for the one parent who was participating in the study. UPPS-P and UPPS-P-C scores were z-scored prior to entering them into analyses.

### 2.3. MRI data acquisition

Scans were conducted using a 3-Tesla Siemens Prisma MRI scanner with a 32-channel head coil. Co-registration and normalization of functional image volumes to Talairach space were completed using a high-resolution 3D magnetization-prepared rapid gradient echo (MPRAGE; 160 sagittal slices; 1.05 × 1.05 × 1.2 mm voxel dimension) scan. For BART runs, we used a T2^*^-weighted gradient echo-planar imaging (EPI) sequence (54 axial slices; voxel size 2.5 × 2.5 × 2.5 mm; TR/TE 1,200/29 ms, flip angle 65°; Field-of-view:220 × 220 mm, Matrix: 88 × 88).

### 2.4. MRI data preprocessing

To reduce noise, individual time points with high motion (>0.5 mm framewise displacement) and/or noise (>10% of voxels considered time-series outliers; AFNI command 3dToutcount) were removed. Individual trials were also removed if reaction times took longer than 5,000 ms. Additionally, entire runs were excluded if motion related outliers took >10% of the time points or >10% of trials took over 5,000 ms in each run. Based on the aforementioned criteria, participants (*n* = 51) with < 2 satisfactory runs were excluded from the analyses. Finally, in line with our previous work (Hulvershorn et al., 2015), subjects who had < 5 explosions across all runs were excluded as it may signal an over-cautious or strategized approach (*n* = 4).

### 2.5. MRI data first-level GLMs

Choice events were modeled as the time at which a participant presses the button to either inflate the balloon (Choose Inflate) or stop inflating and win the money (Choose Win). Outcome events were aligned to the time point when the balloon was successfully inflated (Outcome Inflate) or exploded (Outcome Explode). We did not include the Outcome Win, the time point at which money is banked, in the analyses as there was no uncertainty at this point.

The balloon explosion probabilities at each pump used in the parametric modulation analysis were as follows: 0% for $0.0; 2.1% for $0.05; 4.2% for $0.15; 6.3% for $0.25; 14.6% for $0.55; 23.9% for $0.95; 31.3% for $1.45; 43.8% for $2.05; 56.3% for $2.75; 68.8% for $3.45; 79.2% for $4.25; 89.6% for $5.15.

### 2.6. Statistical analysis

First, the three runs were concatenated prior to first-level modeling. Six motion parameters, six motion derivatives, and detrending terms to correct for scanner drift were modeled. Regressors were created by convolving the train of stimulus events with a double-gamma hemodynamic-response function with a first-order temporal derivative to create a model Blood Oxygenation Level Dependent (BOLD) response time series for each condition. The task-related regressors included five event regressors: two choices (choose inflate, choose win), three outcomes (outcome win, outcome inflate, outcome explode); and a nuisance regressor (choice trials with reaction times >5,000 ms). The task-related regressors were parametrically modulated by the balloon explosion probability for each pump. This approach has been used by multiple groups (Claus et al., 2011, 2013; Hulvershorn et al., 2015; Dir et al., 2020) and allows for examination of reward sensitivity vs. risk-taking behavior.

For specific regions of interest (ROIs), FreeSurfer parcellation from the Desikan-Killiany atlas was used to define ROIs related to the choice conditions (lOFC, rACC, and aIC) and to the outcome conditions (vmPFC, right IFG, and NAcc). These ROIs were selected based on prior work that has identified impairments in these brain regions in high-risk children relative to controls in the BART (Hulvershorn et al., 2015). Laterality was included as a variable of no interest. For further information regarding first-level GLM modeling (see Supplementary material).

For the group analyses, a 3 (Group: HC, PC, HR)-by-2 (Laterality: Left, Right)-by-3 (Region: lOFC, rACC, aIC)-by-2 (Choice: Inflate, Win) repeated measures ANCOVA was conducted on the choice phase data [BOLD response modulated by P^*^ (explode)]. A 3 (Group: HC, PC, HR)-by-4 (Region: vmPFC, right iFG, right NAcc, left NAcc)-by-2 (Outcome: Explode, Inflate) repeated measures ANCOVA was conducted on the outcome phase data [BOLD response modulated by P^*^ (explode)]. In both cases, Parent UPPS-P was included as a covariate of interest as a measure of overall parent impulsivity. Parent UPPS-P was calculated by summing all the subscales of the Parent UPPS-P. To control for child impulsivity (see below), Child UPPS-P was included as a control variable. Child UPPS-P was calculated by adding up all the subscales of the Child UPPS-P. Since male gender was associated with increased parent impulsivity (see below), gender was included as a control variable. We chose this approach as our primary approach because the BOLD responses in our ROIs were significantly correlated, and so such an analysis would not meet the assumptions for a Bonferroni Correction (i.e., no correlations in our dependent variable). Since we did not perform a whole-brain or small volume voxel-wise analysis to identify significant clusters (instead extracting the average parameter estimate from all voxels of our structurally defined ROIs), using cluster correction techniques (i.e., Cox et al., 2016) to correct for multiple comparisons would not be applicable to the current analysis. In order to rule out multiple-comparisons concerns we repeated our ANCOVAs on each individual ROI applying a Bonferroni Correction (see Supplementary material for this analysis).

## 3. Results

In total, 125 participants were included in the study (HR = 50, PC = 47, HC = 28). Males had parents with higher impulsivity ratings [*t*_(123)_= 2.03, *p* < 0.05] but child impulsivity did not differ between males and females [*t*_(123)_= 1.85, ns], across the entire sample (Table 1). Therefore, gender was included as a covariate of no interest in all analyses. As expected, lower impulsivity ratings were observed in the HC group for both children and parents (Table 2). The relationship between total parental UPPS-P and child UPPS-P scores was significant *r*_(125)_= 0.18, *p* < 0.05. Therefore, child UPPS-P was included as a covariate of no interest in all analyses. For further details (see Tables 1, 2 and Supplementary material).

**Table 1:** Sample characteristics by group.

	**Total (*****n*** = **125)**		**HR (*****n*** = **50)**		**PC (*****n*** = **47)**		**HC (*****n*** = **28)**		**HR vs. PC vs. HC**		**HR vs. PC**	
									**Statistics**	***P*** **-values**	**Statistics**	***P*** **-values**
**Age**, M (SD)	11.92	(0.54)	11.98	(0.53)	11.83	(0.52)	11.96	(0.61)	F = 0.98	0.378		
**Sex**, ***n*** **(%)**												
Male	81	(64.80)	32	(64.00)	31	(65.96)	18	(64.29)	χ^2^ = 0.04	0.978		
Female	44	(35.20)	18	(36.00)	16	(34.04)	10	(35.71)				
**Race/ethnicity**, ***n*** **(%)**^**†**^												
C	69	(55.20)	28	(56.00)	27	(57.45)	14	(50.00)	χ^2^ = 0.72	0.949		
>1	23	(18.40)	9	(18.00)	9	(19.15)	5	(17.86)				
AA	33	(26.40)	13	(26.00)	11	(23.40)	9	(32.14)				
**IQ**, M (SD)	108.35	(14.07)	107.04	(14.10)	108.36	(15.12)	110.61	(12.28)	F = 0.57	0.568		
**Parent education**, ***n*** **(%)**^**†**^												
HS or lower	18	(14.63)	9	(18.37)	5	(10.87)	4	(14.29)	χ^2^ = 9.28	0.054		
Higher than HS, lower than Bachelor's degree	66	(53.66)	31	(63.27)	25	(54.35)	10	(35.71)				
Bachelor's degree or higher	39	(31.71)	9	(18.37)	16	(34.78)	14	(50.00)				
**Pubertal development**, ***n*** **(%)**												
Stage 1	38	(30.40)	15	(34.09)	13	(28.89)	10	(38.46)	χ^2^ = 2.39	0.959		
Stage 2	52	(41.60)	19	(43.18)	22	(48.89)	11	(42.31)				
Stage 3	24	(19.20)	9	(20.45)	10	(22.22)	5	(19.23)				
Stage 4	1	(0.80)	1	(2.27)	0	(0.00)	0	(0.00)				
**ADHD**, ***n*** **(%)**^**a†**^												
Other specified ADHD	11	(11.34)	8	(16.00)	3	(6.38)	0	(0.00)			χ^2^= 2.64	0.501
Inattentive type	41	(42.27)	20	(40.00)	22	(46.81)	0	(0.00)				
Hyperactive/Impulsive type	3	(3.09)	1	(2.00)	2	(4.26)	0	(0.00)				
Combined type	42	(43.30)	21	(42.00)	20	(42.55)	0	(0.00)				
**Disruptive behavior disorder type**, ***n*** **(%)**^**a**^												
Conduct disorder^†^	4	(3.20)	1	(2.33)	3	(6.98)	0	(0.00)			χ^2^ = 1.05	0.616
Oppositional defiant disorder	64	(51.20)	32	(78.05)	32	(74.42)	0	(0.00)			χ^2^ = 0.15	0.800
Disruptive mood dysregulation disorder^†^	2	(2.30)	0	(0.00)	2	(4.55)	0	(0.00)			χ^2^ = 2.00	0.494
Other disruptive behavior disorder	18	(21.18)	11	(26.19)	7	(16.28)	0	(0.00)			χ^2^ = 1.25	0.299
**Lifetime psychiatric diagnoses**, ***n*** **(%)**^**b**^												
Depressive disorders^†^	3	(3.45)	2	(4.65)	1	(2.27)	0	(0.00)			χ^2^ = 0.37	0.616
Anxiety disorders^†^	7	(8.05)	6	(13.95)	1	(2.27)	0	(0.00)			χ^2^ = 4.01	0.058
Post-traumatic stress disorders^†^	6	(6.90)	5	(11.63)	1	(2.27)	0	(0.00)			χ^2^ = 2.96	0.110
Adjustment disorders^†^	7	(8.05)	3	(6.98)	4	(9.09)	0	(0.00)			χ^2^ = 0.13	1.000
**Lifetime ADHD medication**, ***n*** **(%)**^**†**^												
Stimulant	30	(30.93)	14	(28.00)	16	(34.04)	0	(0.00)			χ^2^ = 4.54	0.288
Non-stimulant	2	(2.06)	1	(2.00)	1	(2.13)	0	(0.00)				
More than 1	21	(21.65)	8	(16.00)	13	(27.66)	0	(0.00)				
No medication	39	(40.21)	25	(50.00)	14	(29.79)	0	(0.00)				

HR, high risk; PC, psychiatric control; HC, healthy control; M, mean; SD, standard deviation; n, frequency; %, percentage; C, Caucasian; >1, more than one race/ethnicity; AA, African American; HS, high school.

^a^Rates are for current ADHD and disruptive behavior disorder diagnoses.

^b^Rates are for lifetime history (current and past) of psychiatric diagnoses.

^†^Fisher's exact test was conducted.

**Table 2:** Parental and Child UPPS-P.

	**Total (*****n*** = **125)**		**HR (*****n*** = **50)**		**PC (*****n*** = **47)**		**HC (*****n*** = **28)**		**HR vs. PC vs. HC**		**HR vs. PC**	
									**F-statistics**	***p*** **-value**	**t-statistics**	***p*** **-value**
**UPPS-P impulsivity traits (Parent), M (SD)**												
Sensation seeking	2.31	(0.59)	2.36	(0.61)	2.30	(0.58)	2.20	(0.60)	0.54	0.585		
Lack of planning	1.72	(0.46)	1.78	(0.48)	1.76	(0.45)	1.56	(0.40)	2.23	0.112		
Lack of perseverance	1.75	(0.50)	1.87	(0.57)	1.79	(0.46)	1.49	(0.33)	5.37	0.006	0.67	0.416
Negative urgency	2.09	(0.69)	2.30	(0.65)	2.13	(0.68)	1.66	(0.56)	8.16	0.001	1.82	0.181
Positive urgency	1.52	(0.54)	1.66	(0.61)	1.52	(0.48)	1.23	(0.35)	6.28	0.003	1.96	0.165
Total	9.39	(1.98)	10.00	(2.00)	9.48	(1.95)	8.16	(1.43)	8.72	< 0.001	1.65	0.202
**UPPS-P impulsivity traits (Child), M (SD)**												
Sensation seeking	2.76	(0.65)	2.72	(0.62)	2.76	(0.69)	2.81	(0.65)	0.14	0.872		0.713
Lack of planning	2.07	(0.53)	2.05	(0.37)	2.22	(0.55)	1.79	(0.36)	6.07	0.003	1.27	0.262
Lack of perseverance	1.98	(0.38)	2.06	(0.37)	2.00	(0.42)	1.82	(0.29)	3.60	0.030	0.37	0.545
Negative urgency	2.31	(0.69)	2.38	(0.64)	2.45	(0.70)	1.94	(0.64)	5.83	0.004	0.27	0.603
Positive urgency	2.35	(0.75)	2.36	(0.74)	2.55	(0.71)	1.99	(0.77)	5.31	0.006	1.84	0.179
Total	11.47	(1.99)	11.60	(1.97)	11.99	(1.89)	10.35	(1.80)	6.76	0.002	1.00	0.319

HR, high risk; PC, psychiatric control; HC, healthy control; M, mean; SD, standard deviation; n, frequency; %, percentage; UPPS-P, impulsive behavior scale.

### 3.1. BART performance

There were no significant group differences in BART performance variables, except for reaction times (Table 3) where there was a significant Group-by-Choice interaction effect [*F*_(2, 118)_ = 4.59, *p* < 0.05]. The HR group showed a significantly shorter response time for inflate relative to Choose Win decisions compared to the HC group [*t*_(76)_ = −2.46, *p* < 0.05]. The HR group also showed a trend-level shorter response time for inflate relative to Choose Win decisions compared to the PC group [*t*_(95)_ = −1.63, *p* = 0.10]. There were no main effects, or interactions with, parent UPPS-P or child UPPS-P.

**Table 3:** BART behavioral patterns by group.

	**Total (*****n*** = **125)**		**HR (*****n*** = **50)**		**PC (*****n*** = **47)**		**HC (*****n*** = **28)**		**F-Statistic**	***p*-value**
	**M**	**SD**	**M**	**SD**	**M**	**SD**	**M**	**SD**		
**Choice phase**										
Number of pumps (choose inflate)	257.08	41.43	256.41	44.10	260.81	38.60	251.86	42.17	0.44	0.649
Number of stops (choose win)	36.25	12.99	37.22	14.11	36.21	11.46	34.61	13.70	0.32	0.727
**Outcome phase**										
Number of explosions (outcome explode)	17.86	5.98	17.67	6.24	17.88	5.42	18.18	6.62	0.05	0.948
Number of inflations (outcome inflate)	239.22	38.19	238.73	40.15	242.94	35.93	233.68	39.08	0.55	0.581
**Reaction time (ms)**										
Choose inflate	908.53	305.09	882.64	240.85	886.72	296.51	991.21	402.67	1.36	0.261
Choose win	789.48	267.23	839.84	287.35	746.64	241.78	774.79	267.06	1.76	0.177

HR, high risk; PC, psychiatric control; HC, healthy control; M, mean; SD, standard deviation.

### 3.2. BART imaging results

#### 3.2.1. Interactions with group

**Figure 2:**
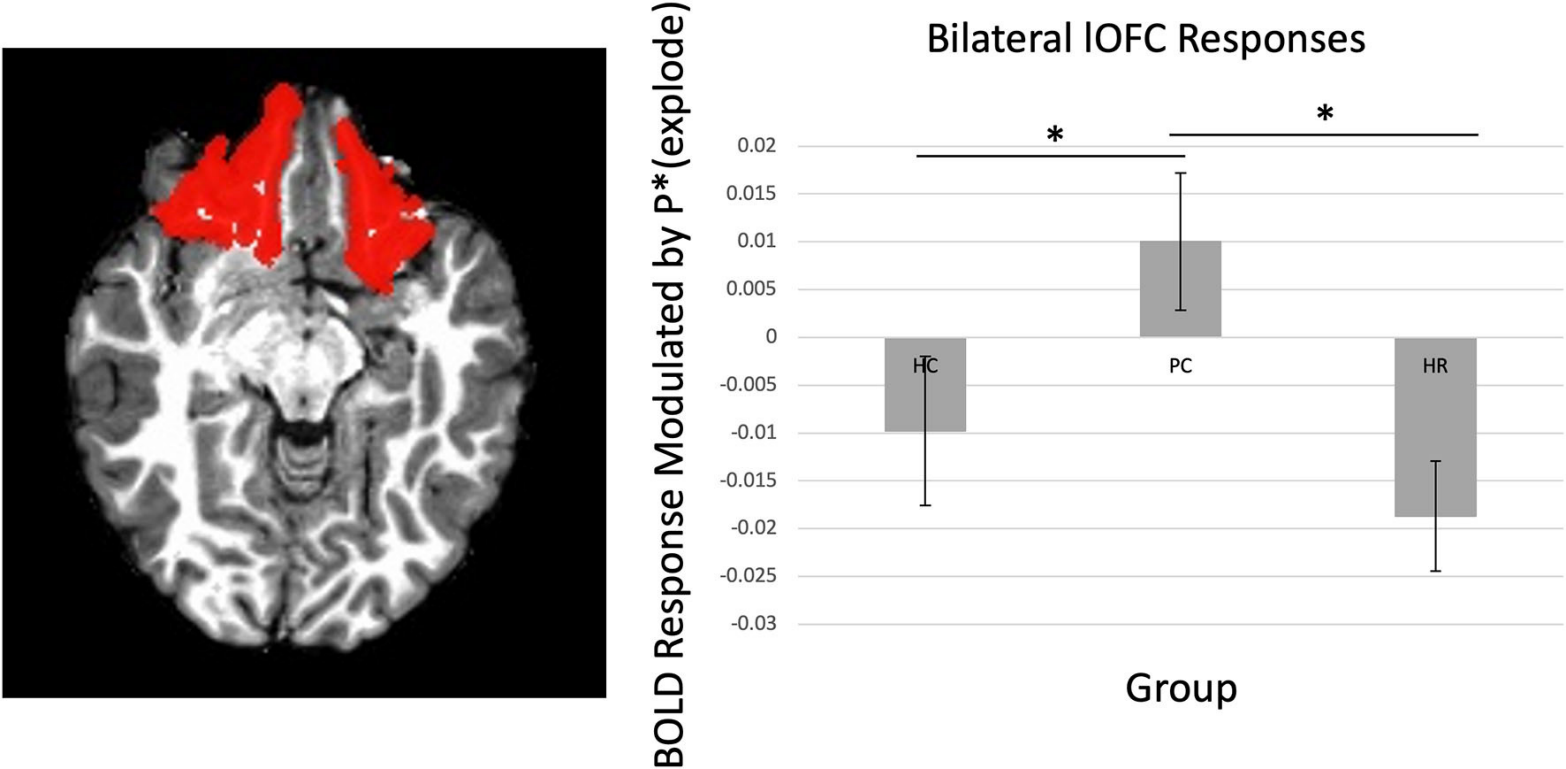
Main effect of group on lOFC. There was increasing activation in the lOFC as balloon explosion probability increased in the Psychiatric Control group relative to the Healthy Control and High-Risk groups. lOFC ROI dilated by one voxel for visualization purposes, see Supplementary material for original mask. ^*^Indicates significant at *p* < 0.05.

There was a significant Group-by-Region interaction effect within the lOFC (Figure 2) [*F*_(4, 236)_ = 2.56, *p* < 0.05]. *Post-hoc* testing revealed that the PC group showed greater BOLD response as balloon explosion probability increased compared to the HC or HR groups within the lOFC [*t*s = 2.06–3.08, *p*s < 0.05].

There was a significant Group-by-Laterality-by-Region-by-Choice interaction [*F*_(4, 236)_ = 2.54, *p* < 0.05]. Follow-up testing revealed a significant Group-by-Laterality-by-Choice interaction within the lOFC, such that individuals in the PC group showed greater BOLD response as balloon explosion probability increased on the right side relative to left side [*t*_(46)_ = 2.42, *p* = 0.02], but not individuals in the HC or HR groups [*t*s < 1.17, *p*s > 0.10].

#### 3.2.2. Interactions with parent UPPS-P

**Figure 3:**
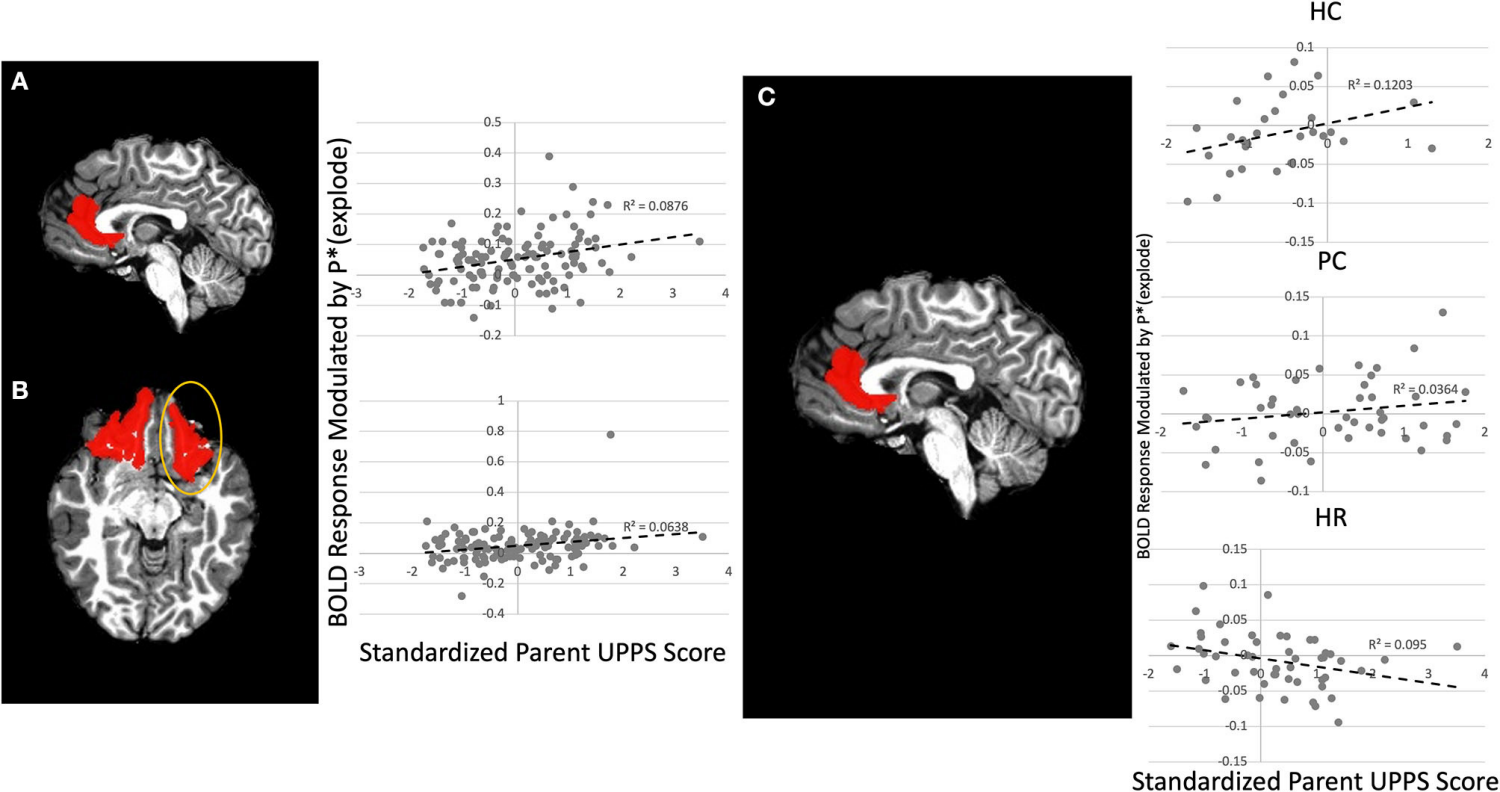
Main Effect of Parental UPPS on left rACC and right lOFC and Group-by-Parental UPPS interaction effect on left rACC. Greater parental UPPS was associated with increasing activation as balloon explosion probability increased in the **(A)** left rACC and **(B)** right lOFC across the entire sample. **(C)** In the High-Risk group parental UPPS was inversely associated with increasing activation as balloon explosion probability increased within the left rACC. rACC and lOFC ROIs dilated by one voxel for visualization purposes, see Supplementary material for original mask.

There was a significant Parent UPPS-P-by-Laterality-by-Region-by-Choice interaction (Figure 3) [*F*_(2, 236)_ = 3.45, *p* < 0.05]. Follow-up testing revealed that Parent UPPS-P was correlated with greater BOLD response as balloon explosion probability increased during inflate choices relative to win choices within right lOFC and left rACC [*r*s = 0.27, *p*s < 0.005] across the entire sample. When taking group into account, there was a significant Group-by-Parent UPPS-P-by-Laterality-by-Region interaction effect (Figure 3C) [*F*_(4, 236)_ = 2.89, *p* < 0.05], such that parent UPPS-P was inversely associated with BOLD response modulation as balloon explosion probability increased within left and right rACC [*r*s = −0.29 to 0.32, *p*s < 0.05]. Within the PC group, Parent UPPS-P was correlated with greater BOLD response modulation as balloon explosion probability increased during within right lOFC only [*r* = 0.29, *p* < 0.05]. Within the HC group, Parent UPPS-P was not correlated with greater BOLD response modulation as a function of increased risk of explosion during inflate choices relative to win choices in any brain region [*r*s = 0.04–0.32, *p*s > 0.10]. None of these results significantly interacted with gender, indicating no significant differences in these interaction effects between boys and girls [*F*s < 0.99, *p*s > 0.05].

#### 3.2.3. Outcome phase

No group differences, or interactions with Parent UPPS-P, were detected within any ROI.

## 4. Discussion

The goal of the current study was to investigate (i) differences in neural functioning during risky decision-making in children with externalizing disorders with and without family histories of SUDs, as well as healthy controls and (ii) the relationship between parental impulsivity and neural functioning during risky decision-making. Unexpectedly, we found that compared to the other groups, individuals with externalizing disorders *without* family histories of SUDs (i.e., psychiatric controls; PC) showed increased activation during riskier choices (i.e., choices with higher probability of balloon explosion), in the lOFC than either of the other groups. These HR and PC groups did not differ on demographics or measures of impulsivity, traumatic events, intellect, socioeconomic status, or risky decision making, but did differ in the presence of familial SUDs. We also report that across all groups, parental impulsivity was associated with brain activation, again as a function of risk of balloon explosion, within the lOFC and rACC while children were making choices. Finally, we found that within the HR group only, parental self-reported impulsivity was inversely associated with activation within rACC, again when children were making choices. However, contrary to our hypotheses, neither group nor parent impulsivity were associated with differences in brain activation during riskier choices when subjects learned the outcomes of their choices, as in our prior study.

Our first finding was increasing activation in the lOFC as balloon explosion probability increased during the choice contrasts in the PC, compared to the control and the HR groups. Group differences were not observed in any other brain region. Animal research suggests that the OFC is involved with integration of choice, prior information, and stimulus information when making choices (Nogueira et al., 2017) and orchestrating the attentional response to potentially rewarding stimuli during action selection (Cho et al., 2016). Specifically, the *lateral* OFC plays a role in associative learning processes that link stimuli with reward values, while the *medial* OFC plays a role in encoding expected values of stimuli (Noonan et al., 2010), and this finding has been replicated in humans with lateral vs. medial OFC lesions (Noonan et al., 2017). In human neuroimaging studies, greater OFC activity has been associated with risky vs. safer decision-making (Ernst et al., 2002). It should be noted that there were no differences between the HR and control groups in lOFC activation as balloon explosion probability increased. This is in contrast to prior work indicating that children with co-morbid conduct problems and drug-using behaviors show reduced activity in these brain regions during risky decision-making compared to controls (Crowley et al., 2010). One possibility is that the neural correlates of risk for substance use behaviors are distinct from neural correlates of substance use behaviors in children and adolescents. Future longitudinal work will be necessary to investigate this possibility. In short, we suggest that impaired function in the lOFC indicates difficulty in linking stimuli with reward in youth with externalizing psychopathology. However, this is not observed in children from families with SUDs, perhaps related to other uncharacterized abnormalities in circuitry that render the OFC unimpacted.

Our second finding was that parental impulsivity was associated with greater activation within the entire sample of children's right lOFC and left rACC as balloon explosion probabilities increased. However, within the rACC, the relationship between parental impulsivity and BOLD response modulation differed by group, such that in the HR group, parental impulsivity was associated with reduced BOLD response as balloon explosion probability increased during the choice phase. The rACC is a brain region that is involved with representation and integration of subjective value of stimuli, based on prior experiences (Walton et al., 2007; Blair et al., 2013) as well as in encoding of affective stimuli (Blair et al., 2013) and errors (Forman et al., 2004). Although impulsivity and externalizing behaviors are heritable (Young et al., 2009; Tiego et al., 2020), it has been speculated that there may be different combinations of neuro-circuitry dysfunction that give rise to impulsive behaviors, including externalizing and/or substance use behaviors, in adolescents (Parvaz et al., 2018; Ivanov et al., 2019). A meta-analysis showed that in healthy individuals, impulsivity was directly associated with reward system activation during reward anticipation, but in individuals with high levels of externalizing psychopathology, impulsivity was inversely associated with reward system activation during reward anticipation (Plichta and Scheres, 2014). Adolescents with co-morbid externalizing disorders and FH of SUDs show differences in neuro-computational parameters underlying risky decision-making compared to adolescents with externalizing disorders (Parvaz et al., 2018; Ivanov et al., 2019), indicating that the neurobehavioral mechanisms underlying risky decision-making may be intergenerationally transmitted. As there seem to be multiple neurobehavioral mechanisms underlying risky decision-making (Plichta and Scheres, 2014), our data suggests that some of these neurobehavioral mechanisms underlying risky decision-making are transmitted from parent to child. However, it should be noted that males had greater parental impulsivity than females. While we did covary for gender in our analysis, prior work has shown that genetic variance is more strongly associated with externalizing behaviors in males relative to females (Hicks et al., 2007); given evidence for genetic transmission of impulsivity (Tiego et al., 2020), we speculate that the heritability of neurocognitive factors associated with impulsivity (and ultimately, externalizing behaviors) is stronger in males than females. Future work will be necessary to disentangle whether these relationships between parental impulsivity and brain activity and behavior are heritable, influenced by environment, or both as well as the degree to which this may be moderated by gender.

The striatum and various regions of prefrontal cortex such as OFC and ACC are targets of dopaminergic projections involved in reward processing and are involved in encoding value of reinforcing stimuli (O'Doherty et al., 2017). However, the relationship between reward processing and addiction risk in children and adolescents is complex. It has been suggested that striatal activation during reward anticipation is inversely associated with risk-taking in adolescents (Schneider et al., 2012). However, previous work on the role of reward processing and risk for adolescent substance use has been mixed. Although some studies have shown that *increased* activation to reward anticipation predicts future substance use in adolescents (Heitzeg et al., 2014) other studies indicate that *reduced* activation within striatum and prefrontal cortex during reward anticipation predict future substance use (Büchel et al., 2017). Notably, the sample from Büchel et al. (2017) had elevated impulsivity. It has been suggested that neural risk factors for risky behaviors, including substance use, may differ between populations with and without psychopathology. More specifically, theoretical models of externalizing behavior have suggested that increased activity in these circuits is associated with increased risk in healthy populations while decreased activity in these circuits is associated with increased risk in populations with underlying psychopathology, such as ADHD (Plichta and Scheres, 2014). The current data indicate that there is a significant main effect of parental impulsivity on brain activity within lOFC and rACC during risky decision-making but that this relationship is moderated by externalizing psychopathology.

Contrary to our hypotheses, there were no effects of group or parental impulsivity on BOLD response modulation in the outcome phase of the task. This is inconsistent with our prior work in a smaller sample, which showed that individuals with co-morbid family histories of SUD and externalizing disorders showed greater activation within ventral striatum, vmPFC, and aIC during explode outcomes, depending on balloon explosion probability (Hulvershorn et al., 2015). One possible explanation for this discrepancy is that the task in the current study is substantially longer than in prior work (24 vs. 8 min) to increase the number of trials per participant and improve the overall design (Chen et al., 2022). It is possible that some of the findings reported previously were due to Type II error due to the shorter scan time (and lower number of trials per participant) in previous work. It is also possible that there are some differences in the prior sample vs. the current sample contributing to this discrepancy. Ninety-six percent of children in the HR group in the prior sample were diagnosed with ADHD (Combined type) whereas only 42% of children in the HR group in the current sample carried the same diagnosis (i.e., a higher proportion of predominantly inattentive ADHD).

There are several limitations to this study. First, participants in the current study have not initiated substance use behaviors at the time of the scan so it is impossible at this time to determine whether the current data represent risk for substance use behaviors above and beyond the clinical history and family history components. Although prior literature indicates that both externalizing disorders (Moss and Lynch, 2001) and family history of SUDs (Iacono et al., 2008) confer risk for the development of substance use behaviors, follow up will be necessary after these individuals have begun to engage in substance use in order to determine whether these brain level differences represent risk for substance use behaviors above and beyond clinical/family history. Second, the PC group had a slightly greater proportion of individuals who had been treated with psychotropic medications in their lifetimes; therefore, it is possible that differences across exposure to prescribed psychotropic medications (particularly stimulants) may have played a role in the current findings. Third, we did not consider the potential heterogeneity in externalizing symptoms (ADHD vs. CD vs. ODD) or severity of symptoms across groups in our analysis. Although the PC and HR groups were matched on frequency of ADHD, CD, and ODD diagnoses and UPPS-P scores did not differ, it is possible that either the PC or HR groups may have differed on symptom severity that we were unable to measure which may have influenced the current results. Future investigation will be necessary to delineate these possibilities. Also, although it can be argued that we conducted multiple tests, warranting corrections for multiple comparisons it should be noted that we utilized a repeated measures design for our main analysis of interest, mitigating this concern. We repeated our analysis running each ROI individually and applying a Bonferroni correction for six ROIs for the choice phase and four ROIs for the outcome phase (rather than coding them as within-subjects variables in our ANCOVA models); the rostral ACC findings were significant at a Bonferroni-corrected threshold, however, the lOFC findings were significant only at uncorrected thresholds (see Supplementary material for further information). Additionally, the HR group had greater prevalence of mood and anxiety disorders compared to the other groups. Although this difference only approached statistical significance in anxiety disorders, we re-ran the analysis removing individuals with mood and anxiety disorders, and this did not significantly change our results. Finally, impulsivity is a heterogeneous construct, consisting of multiple personality traits, such as positive urgency, negative urgency, and sensation seeking, among others (Zapolski et al., 2010). Future work should investigate the extent to which these individual traits are heritable, and if so, to what extent they are associated with alterations in reward processing neuro-circuitries in children at risk for SUDs.

In summary, we found that subjects with externalizing disorders without FH of SUDs (PC) showed greater lOFC modulation during the choice phase of the BART than other carefully matched groups, suggesting SUD risk mechanisms related to reward learning that may be distinct in youth without familial SUD. Interestingly, parental impulsivity was independently associated with greater modulation within the childrens' rACC and lOFC across the entire sample. Further, our data point to a potential biomarker associated with parental impulsivity in the familiar HR group within the rACC, suggesting core deficits in representation and integration of the value of potential rewards could be important targets for preventive interventions. In total, these data indicate that the relationship between parental impulsivity and integrity of neuro-circuitries underlying decision-making may be different between individuals with and without FH of SUDs, particularly in prefrontal cortical regions central to decision making. These findings suggest that biomarkers of SUD risk may be different in adolescents with and without FH of SUDs; future work should follow samples longitudinally to examine whether certain biomarkers are effective at predicting SUD development in these populations. We propose that development of such biomarkers would provide further insight into determining level of risk for SUDs across different populations.

## Data availability statement

The data detailed in this paper are part of an ongoing longitudinal study and will be made public at the conclusion of the study. Until then, the data that support the findings of this study are available from the corresponding author (LH) upon reasonable request. Requests to access the datasets should be directed to LH; lhulvers@iu.edu.

## Ethics statement

The studies involving human participants were reviewed and approved by IUPUI Institutional Review Board. Written informed consent to participate in this study was provided by the participants' legal guardian/next of kin.

## Author contributions

JA, EK, and LH were responsible for study conception, study design, data analysis and interpretation, and drafting of the manuscript. TH was involved in data analysis. KC, MA, PF, and JN were involved in data interpretation. NS and LP were involved in data acquisition. All authors were involved in critical revision of the manuscript and provided approval of the manuscript prior to submission.
