# Supplementary material for: Family history of substance use disorder and parental impulsivity are differentially associated with neural responses during risky decision-making
Source: Front Neuroimaging. 2023 Jun 21;2:1110494. doi: 10.3389/fnimg.2023.1110494 (PMC10406275; doi:10.3389/fnimg.2023.1110494)
Supplement: Supplementary file 1 [file Data_Sheet_1.docx]

***Supplemental Material***

**Family History of Substance Use Disorder and Parental Impulsivity are Differentially Associated with Neural Responses During Risky Decision-Making**

Joseph Aloi, MD, PhD^1^, Elizabeth Kwon, PhD^1^, Tom A. Hummer, PhD^1^, Kathleen I. Crum, PhD^1^, Nikhil Shah, BS^1^, Lauren Pratt, BS^2^, Matthew Aalsma, PhD^3^, Peter Finn, PhD^4^, John Nurnberger, MD, PhD^1^, Leslie A. Hulvershorn, MD, MSc^1^

**Supplemental Analyses**

We repeated our main analysis for the choice phase in each individual ROI. For the choice phase analyses, a 3 (Group: HC, PC, HR)-by-2 (Choice: Inflate, Win) repeated measures ANCOVA (with Parent UPPS-P as a covariate) was conducted on the choice phase data (BOLD response modulated by P*(explode)) in each of the 6 ROIs (R/L anterior insula, R/L lateral orbitofrontal cortex, R/L rostral anterior cingulate cortex). Regions were considered significant at a Bonferroni-corrected *p*<.05 (uncorrected *p*<.0083).

We repeated our main analysis for the outcome phase in each individual ROI. For the outcome phase analyses, a 3 (Group: HC, PC, HR)-by-2 (Outcome: Inflate, Explode) repeated measures ANCOVA (with Parent UPPS-P as a covariate) was conducted on the outcome phase data (BOLD response modulated by P*(explode)) in each of the 4 ROIs (R/L nucleus accumbens, ventromedial prefrontal cortex, right inferior frontal gyrus).

**Supplemental Results**

As shown in Table 1, age, IQ, parent education, and pubertal development did not differ by group. There were also no correlations between parent UPPS-P/child UPPS-P and age, IQ, parent education, or pubertal development. There was also no relationship between group and sex or ethnicity. Males had parents with higher impulsivity ratings [*t*(123)=2.03, *p*<.05] but child impulsivity did not differ between males and females [*t*(123)=1.85, ns], across the entire sample. Therefore, gender was included as a covariate of no interest in all analyses. There were no differences in parent or child impulsivity between subjects with different ancestries or ethnicities.

Additionally, the two groups with psychopathology (HR and PC) did not differ in impulsivity traits of parents or children, or in lifetime exposure to psychotropic medication use.

***BOLD Response Data***

***Choice Phase***

There were no significant effects of group, Parent UPPS-P, group-by-Parent UPPS-P interaction on BOLD response modulated by risk within left anterior insula (*F*’s<1.2, *p*s>.30). There were no group-by-choice, Parent UPPS-P-by-choice, or group-by-Parent UPPS-P-by-choice interactions BOLD response modulated by risk within left anterior insula (*F*’s<3.41, *p*s>.067).

There were no significant effects of group, Parent UPPS-P, group-by-Parent UPPS-P interaction on BOLD response modulated by risk within left orbitofrontal cortex (*F*’s<1.49, *p*s>.23). There were no group-by-choice, Parent UPPS-P-by-choice, or group-by-Parent UPPS-P-by-choice interactions BOLD response modulated by risk within left anterior insula (*F*’s<0.92, *p*s>.40).

There was a significant effect of group on BOLD response modulated by risk within left rACC (*F*=5.19, uncorrected *p*=.007, Bonferroni-corrected *p*=.042). There was a significant group-by-Parent UPPS-P interaction effect within left rACC (*F*=5.62, uncorrected *p*=.005, Bonferroni-corrected *p*=.03). The main effect of Parent UPPS-P was not significant (*F*=1.39, *p*=0.241). The Parent UPPS-P-by-choice interaction was significant at an uncorrected threshold (*F*=4.52, uncorrected *p*=.036, Bonferroni-corrected *p*=.216). The group-by-choice and group-by-Parent UPPS-P-by-choice interactions were not significant (*F*s<0.5, *p*s>.60).

The group, Parent UPPS-P-and group-by-Parent UPPS-P effects on BOLD response modulated by risk were not significant within right anterior insula (*F*s<0.59, *p*s>0.44). The group-by-choice, Parent UPPS-P-by-choice, and group-by-Parent UPPS-P-by-choice interaction effects were not significant within right anterior insula (*F*s<1.49, *p*s<0.224).

The group-by-Parent UPPS-P interaction effect within right orbitofrontal cortex was significant at an uncorrected threshold (*F*=4.22, uncorrected *p*=.017, Bonferroni-corrected *p*=.10). The main effect of group and the main effect of Parent UPPS-P were not significant (*F*s<2.78, *p*s>.066). The Parent UPPS-P-by-choice interaction was significant at an uncorrected threshold (*F*=5.12, uncorrected *p*=.026, Bonferroni-corrected *p*=0.156).

***Outcome Phase***

No group differences or interactions with Parent UPPS-P were significant at uncorrected thresholds within R/L nucleus accumbens or ventromedial prefrontal cortex

There was a significant effect of group and group-by-UPPS-p interaction effect on BOLD response modulated by P*(explode) within right inferior frontal gyrus (*F*s>6.01, uncorrected *p*s<.003, Bonferroni-corrected *p*s<.012). The main effect of UPPS-p was not significant (F=0.725, *p*=0.40)*.* There was a significant group-by-outcome and group-by-UPPS-p-by-outcome interaction effect (*F*s>4.71, uncorrected *p*s<.011, Bonferroni-corrected *p*s<.044). The UPPS-p-by-outcome interaction effect was not significant.

| 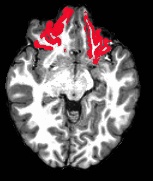 | 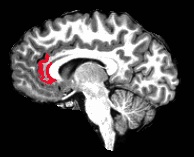 |
| --- | --- |
| Supplemental Figure: Original masks of lOFC (left panel) and rACC (right panel) | |
